# Development of electrically conductive hybrid composites with a poly(lactic acid) matrix, with enhanced toughness for injection molding, and material extrusion-based additive manufacturing

**DOI:** 10.1016/j.heliyon.2022.e10287

**Published:** 2022-08-17

**Authors:** Roland Petrény, Csenge Tóth, Aurél Horváth, László Mészáros

**Affiliations:** a Department of Polymer Engineering, Faculty of Mechanical Engineering, Budapest University of Technology and Economics, Műegyetem rkp 3 H-1111 Budapest, Hungary; b ELKH–BME Research Group for Composite Science and Technology, Műegyetem rkp. 3, H-1111 Budapest, Hungary

**Keywords:** Nanocomposite, Hybrid composite, Carbon nanotubes, Conductive composite, Fused filament fabrication

## Abstract

In this study, we developed electrically conductive nano- and hybrid composites with a poly(lactic acid) (PLA) matrix for different melt processing technologies. We used short carbon fiber and multi-walled carbon nanotube reinforcements to enhance electric conductivity. We prepared the composite compounds with twin-screw extrusion; then the compounds were processed via injection molding and fused filament fabrication. We showed that electric conductivity only slightly increased by when only carbon nanotubes were added to the PLA matrix. However, when carbon fibers were added to the nanocomposites, the higher shear during melt mixing helped the uniform dispersion of the carbon nanotubes, resulting in a highly conductive reinforcement network in the composite. On the other hand, the hybrid reinforcement resulted in higher viscosity, making melt processing difficult and the material also became more brittle. Therefore, we added an oligomeric lactic acid plasticizer to the hybrid composites, and produced specimens by injection molding and 3D printing. The tensile strength increased by 140% and the elongation at break increased by 56%, and at the same time, the electrical conductivity of the material remained at a high level.

## 1 Introduction

The interest in electrically conductive polymers has constantly been growing in recent decades. Dynamically developing sectors such as sensor manufacturing, biomedical applications, and the electronic industry sees their potential, which justifies further research of these materials. Although conductive polymers, such as polyacetylene, polypyrrole, and poly(3,4-ethylene dioxythiophene) have long been known, it is difficult to process them with mass-producing technologies. This justifies the production of conductive polymer composites (CPCs) that can be processed with conventional technologies like melt compounding and injection molding [1, 2, 3]. In this case, conductive particles are dispersed in the insulating polymer matrix. These can form electrically conductive paths, called percolations, thereby increasing the electrical conductivity of the composite [4].

In addition to the electrically conductive polymer composites, there is also a growing interest in biopolymers that can be produced from renewable resources or are biodegradable. These offer a possible alternative to conventional petroleum-based polymers [5, 6, 7]. One of the most popular biopolymers is polylactic acid (PLA), whose monomer can be produced by the fermentation of renewable sources, such as cellulose or other materials containing polysaccharide [8]. Due to its biocompatibility, there is a growing interest in its medical applications: in medical implants, tissue engineering, orthopedic devices, etc. [9]. PLA has poor electrical conductivity (3.32 · 10^−12^ S/cm [8]), similarly to other unfilled polymers, therefore, the use of conductive fillers and reinforcements is intensively researched.

Due to their excellent electrical conductivity (10^5^ S/cm [9]), carbon nanotubes (CNTs) are recently used as conductive fillers. Furthermore, due to their excellent mechanical, physical, and chemical properties, CNT is one of the most researched nanoparticles in recent decades. However, carbon nanotubes should be uniformly dispersed in the matrix to improve conductivity considerably. If they are not dispersed properly, the increment in conductivity is minimal [10]. For example, Wang et al. [11] used poly(ethylene oxide) as a binder for CNTs which helped them to prepare well-dispersed PLA/CNT composites. They showed that the electrical conductivity of the composites improved by two orders of magnitude in case of better dispersion. The importance of dispersion for electrical conductivity is also emphasized in the work of Wang et al. [12], in which an outstanding, 72.2 S/m electrical conductivity have been achieved for PLA/CNT composites.

We investigated the effect of nanoparticles, such as carbon black (CB) and multi-walled carbon nanotubes (MWCNTs) in the PLA matrix. They increased the conductivity of the composites in different degrees (11.3 S/cm for MWCNT/PLA and 0.1 S/cm for CB/PLA prepared by material extrusion-based additive manufacturing [13], and 0.125 S/cm for CB/PLA prepared by hot pressing [14]). *Graphene nanoplatelets* have also been used for PLA matrix in literature with varying results (6.7 · 10^−^^5^ S/cm [11] to 2.42 S/cm [15].

When the nanoparticles are dispersed well, they also increase tensile strength and modulus. However, elongation decreases as the amount of nanoparticles increases, which is not desirable for the inherently brittle PLA [16].

Based on our previous studies, the dispersion of CNT can be significantly improved with the addition of a micro-sized conductive filler, e.g., carbon fiber (CF), during compounding [17, 18, 19]. In this case, other shear forces are formed in the melt due to the presence of CF, which help to disperse the fillers uniformly. In addition to the fact that nanoscale percolations of properly distributed CNTs already significantly increase electrical conductivity, additional microscale percolations are formed through the carbon fibers. Also, there is a synergistic effect between nanoscale percolations produced by CNTs and microscale percolations produced by carbon fibers. Since microscale conductive paths form connections between nanoscale percolations, nanoscale paths form connections between the micro-sized carbon fibers, thereby increasing electrical conductivity [20, 21].

The high viscosity requires higher injection pressure and may lead to a lower degree of mold filling in injection molding or may lead to melt flow instability in the case of extrusion [22]. As there is a growing demand for customizable products, fused filament fabrication has come to the fore, where melt viscosity has an even more important role, due to the very narrow printing nozzle [23]. A commonly encountered defect of composite 3D printing is the clogging of the nozzle, which is often experienced above 20 wt% fiber content [24, 25, 26]. Therefore, the melt viscosity of hybrid composites should be kept at a low level so that the material is melt processable.

In the past few years, oligomeric lactic acid (OLA) has been found to be an effective and environmentally-friendly plasticizer and lubricant for PLA materials. OLA plasticizers help the melt processing of the materials and increase the toughness and elongation at the break of the brittle PLA composites [27, 28].

In this study, we produced electrically conductive polymer composites with a biopolymer matrix, using PLA, CF, and CNT. The hybrid reinforcement has beneficial effects on the dispersion of the nanotubes and leads to an increase in electric conductivity. However, it makes the PLA matrix even more brittle and increases melt viscosity, which makes the melt processing of the material difficult. For this, we use oligomeric lactic acid as a plasticizer, and as a result, the composite should be well processable via injection molding and fused filament fabrication. The goal is to produce an easy-to-process, electrically conductive material for injection molding and material extrusion-based additive manufacturing with enhanced toughness.

## 2 Materials and methods

PLA 4060D amorphous polylactic acid granules manufactured by NatureWorks LLC were used as a matrix material for the composites. The multi-wall CNT used as a nanoscale reinforcement is Nanocyl NC7000 by Nanocyl S. A., with a diameter of 9.5 nm, a length of 1.5 μm, and a specific surface area of 250–300 m^2^/g. Panex 35 Chopped Pellet 95 of Zoltek Zrt. was used as a fibrous reinforcement. As a plasticizer, Condensia Glyplast OLA2 was used.

The fibers had a diameter of 8.3 μm, a length of 6 mm, and a density of 1.81 g/cm^3^. It was necessary to dry the PLA granules before processing. A Faithful WGLL-125 BE drying oven was used to dry the PLA granules for 4 h at 45 °C. The granules and the reinforcing materials were first dry mixed, then compounded with an LTE 26–44 twin-screw extruder manufactured by Labtech Engineering Co., Ltd. Screw speed was 25 rpm and zone temperatures were 180 °C, 190 °C, 190 °C, 190 °C, 190 °C, 200 °C, 200 °C, 200 °C, 200 °C, 200 °C, and 190 °C. The composition of the materials is shown in Table 1.

**Table 1:** Reinforcement content of the composite samples.

Name	PLA (wt%)	CNT (wt%)	CF (wt%)	OLA2 (wt%)
PLA	100.00	0.00	0.00	0.00
PLA+0.25CNT	99.75	0.25	0.00	0.00
PLA+0.5CNT	99.50	0.50	0.00	0.00
PLA+0.75CNT	99.25	0.75	0.00	0.00
PLA+1CNT	99.00	1.00	0.00	0.00
PLA+30CF	70.00	0.00	30.00	0.00
PLA+30CF+0.25CNT	69.75	0.25	30.00	0.00
PLA+30CF+0.5CNT	69.50	0.50	30.00	0.00
PLA+30CF+0.75CNT	69.25	0.75	30.00	0.00
PLA+30CF+1CNT	69.00	1.00	30.00	0.00
PLA+30CF+0.75CNT+10OLA	59.00	1.00	30.00	10.00

The fibers formed during the continuous extrusion were passed through a cooling conveyor belt to an LZ-120/VS type granulator, which produced 4 mm long granules. Before injection molding, the granules were dried as described above. Specimens according to the EN ISO 527-2: 1999 standard were injection molded on an Arburg Allrounder Advance 270S 400-170 injection molding machine with zone temperatures of 185 °C, 190 °C, 195 °C, 200 °C, 200 °C, a mold temperature of 25 °C and an injection pressure of 1500 bar.

**Figure 1:**
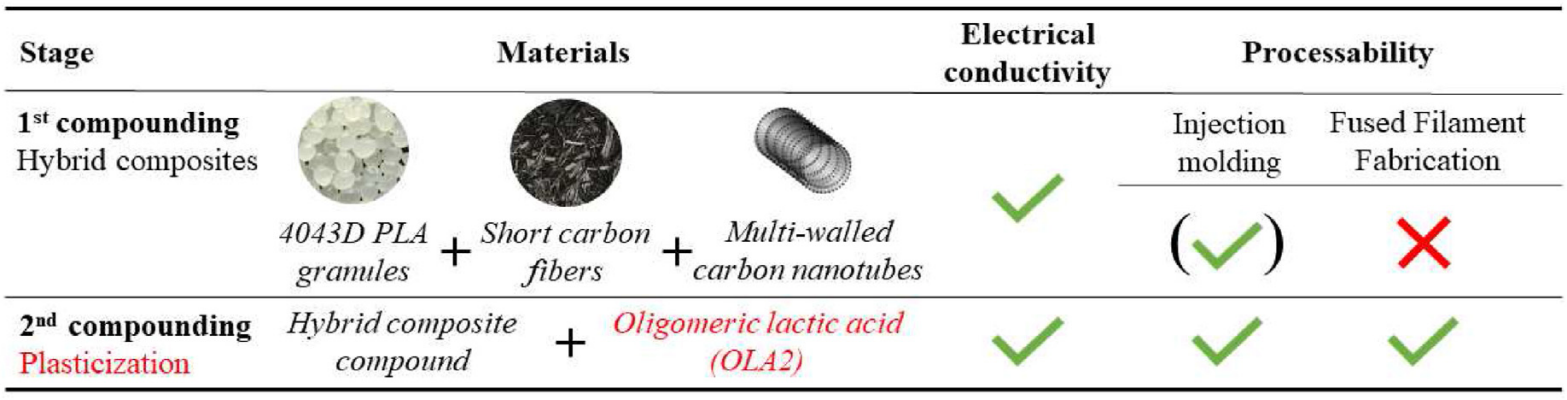
Preparation of the hybrid electrically conductive composites and plasticization for processability.

After determining the optimal mixture for conductivity, processability was improved with an oligomeric lactic acid plasticizer (OLA2). The PLA+30CF+0.75CNT composite was plasticized with 10 wt% OLA2, based on a previous study [29]. To mix the OLA2 with the composite, we fed the PLA+30CF+0.75CNT material into an LTE 26–44 twin-screw extruder, and preheated the OLA2 to 80 °C and dosed it with a Labtech LDF-1.6 liquid dosing system. The zone temperatures in the extruder were 180 °C, 190 °C, 190 °C, 190 °C, 190 °C, 200 °C, 200 °C, 200 °C, 200 °C, 200 °C, and 190 °C, and screw speed was 10 rpm Figure 1 shows a schematic summary of the preparation of the composites.

**Figure 2:**
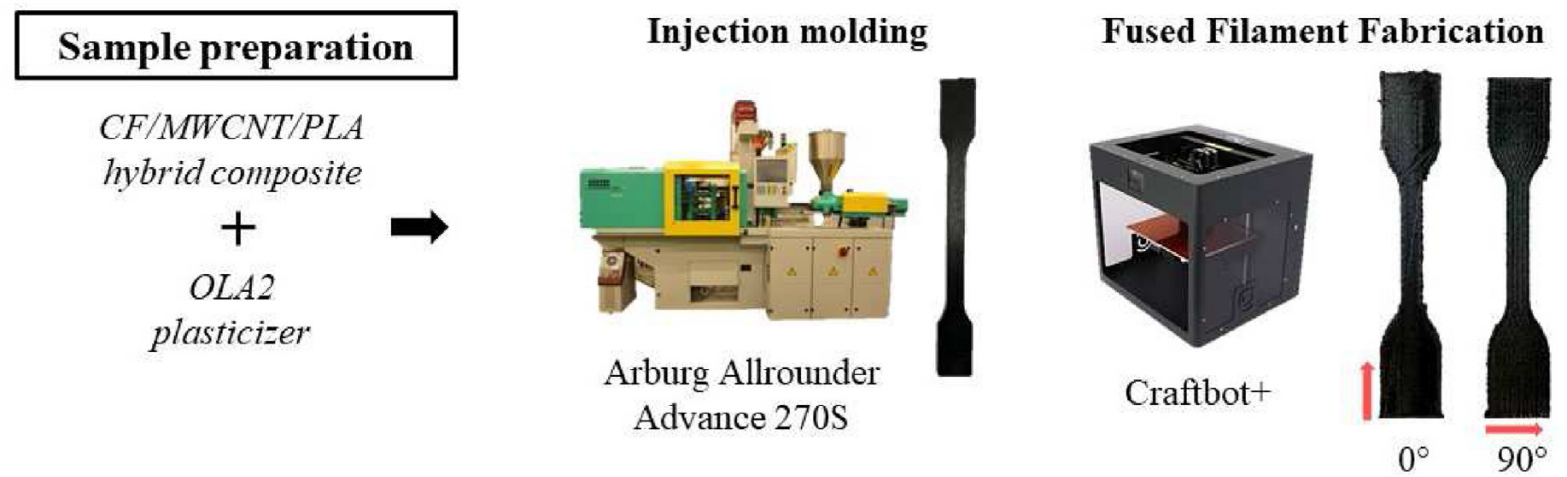
Production of tensile samples by injection molding and fused filament fabrication.

The filament forming during the extrusion had a diameter of 1.65–1.8 mm and was directly applicable for 3D printing. Samples were produced on a Craftbot + desktop material extrusion printer with a nozzle temperature of 220 °C, a layer height of 0.4 mm, and an infill rate of 100%. To investigate the orientation dependence of electrical conductivity and tensile properties, we manufactured two types of specimens with the printing orientation parallel to the longitudinal axis (0°) and perpendicular to it (90°). For the injection molding of the composite plasticized with OLA2, the extruded filament was used after granulating. It was injection molded on an Arburg Allrounder Advance 270S 400-170 injection molding machine with zone temperatures of 185 °C, 190 °C, 195 °C, 200 °C, 200 °C, a mold temperature of 25 °C and an injection pressure of 1500 bar. Figure 2 shows the equipment used and the samples prepared.

The melt flow index (MFI) of the materials was measured on a CEAST 7027.000 capillary plastometer at 200 °C and with a load of 21.6 N. The granules made from the extruded filaments were used for the measurements.

A four-pin resistance meter with an Agilent 34970A data logger was used to measure electrical conductivity. The specific resistance of the composite specimens was determined using Eqs. (1) and (2).

(1) $$\rho =\frac{\text{π·c}}{\text{ln}\left(2\right)}\cdot R\;\left(\Omega cm\right)$$

(2) $$G=\frac{1}{\rho }\;\left(\frac{S}{cm}\right)$$

where *ρ* is the resistivity measured, *c* is the thickness of the sample in cm, *R* is the measured resistivity, and *G* is electrical conductivity.

Tensile tests were carried out on at least five specimens for each material on a Zwick Z005 universal testing machine (Germany) according to EN ISO 527. The tensile moduli were determined with the linear regression line between the 0.05% and 0.25% displacement values. Tensile speed was 2 mm/min, and gauge length was 110 mm.

Density was measured with a Sartorius Quintix 125D type semi-micro scale. At least five samples for each material were tested in water at 22.6 °C.

For the scanning electron microscope (SEM) images, the samples were etched in a 5 mol/l NaOH solution for 1 h at 25 °C and then sputtered with gold. The images were made with a JEOL JSM6380LA scanning electron microscope.

## 3 Results and discussion

### 3.1 Development of electrically conductive hybrid composites

#### 3.1.1 MFI

**Figure 3:**
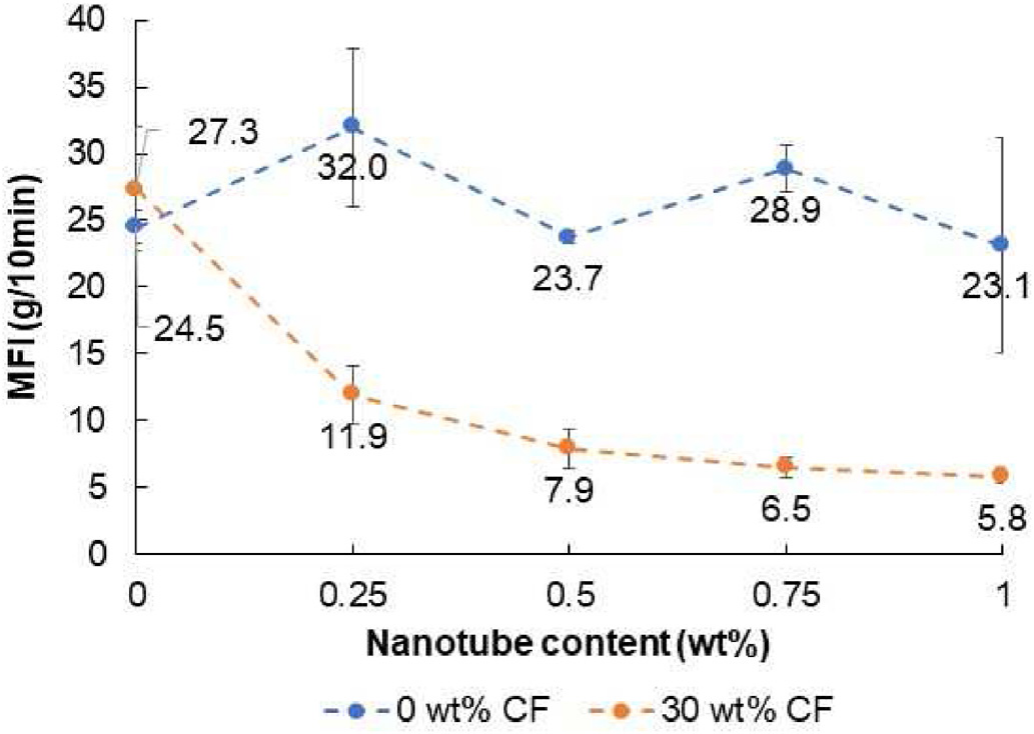
Melt flow index (MFI) of the nano- and the hybrid composites.

The melt flow index of the materials has a key role in their processability. Figure 3 shows that adding only nanotubes to the PLA did not influence its viscosity. However, when 30 wt% carbon fiber was added to it, increasing nanotube content decreased MFI and increased viscosity. If the carbon nanotubes are well dispersed in the matrix, more polymer chains can entangle around them, blocking their movement during melt processing. However, a large MFI makes melt processing difficult or even impossible, especially where low viscosity is required (injection molding, 3D printing).

#### 3.1.2 Electrical conductivity

**Figure 4:**
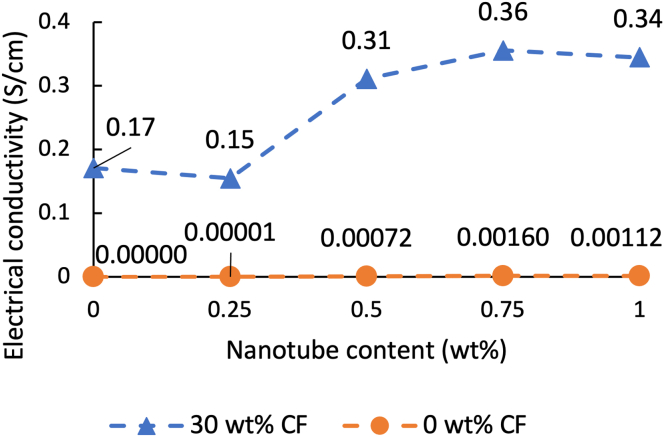
Electrical conductivity of the nano- and the hybrid composites.

The results of the electrical conductivity test are illustrated in Figure 4. The conductivity of the composites reinforced only with CNT remained approximately unchanged. It is due to the aggregation of CNTs, which reduces the number of CNTs involved in the formation of conductive pathways.

**Figure 5:**
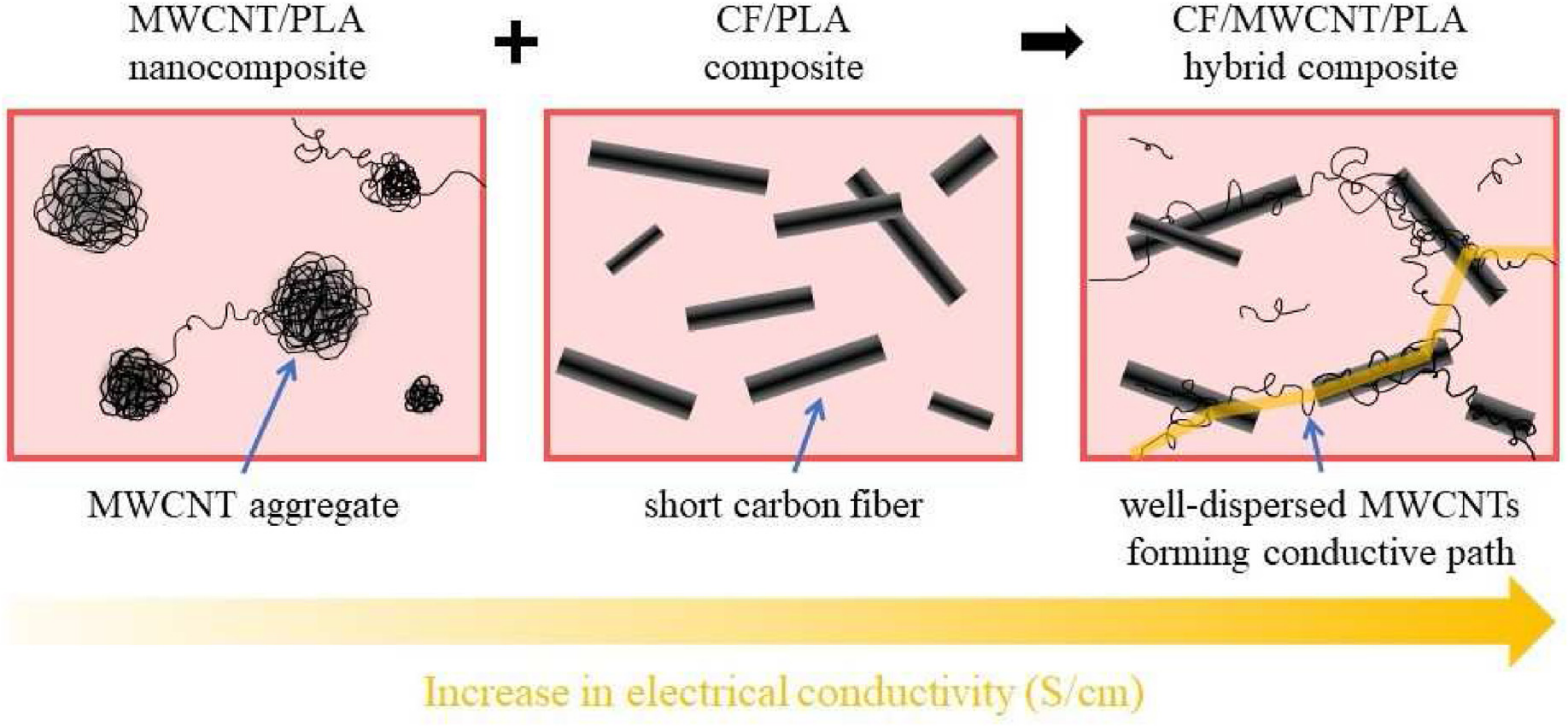
Schematics of the microstructure of the composites and the forming of conductive paths in the hybrid composites.

The conductivity of the hybrid composites reinforced with CNT and CF was already significantly higher than that of the CNT-only composites, due to the presence of 30 wt% CF. With the addition of 0.5 and 0.75 wt% CNT, conductivity increased significantly, reaching twice the conductivity of the CF-only composite (0.355 S/cm). In addition to the nanoscale conductive paths formed by the contact of well-dispersed CNTs, additional microscale tracks are created by the contact of CFs. The two types of reinforcing materials thus help each other's the conductivity, as CFs help to connect the different nanoscale conductive pathways formed by the CNTs. Figure 5 illustrates the development of conductive paths in the hybrid composites.

#### 3.1.3 Tensile properties

**Figure 6:**
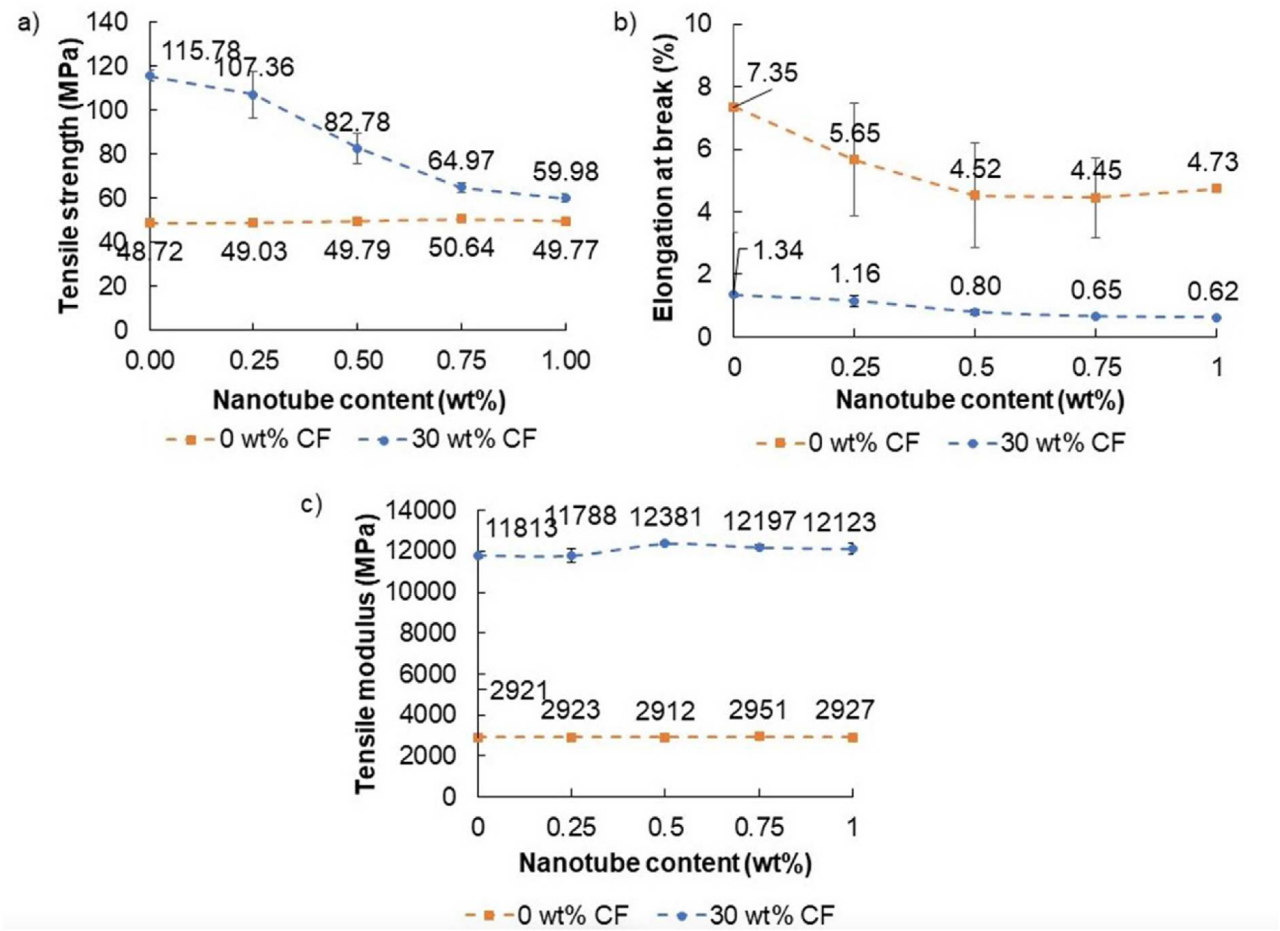
a) tensile strength, b) elongation at break, and c) tensile modulus of the nano- and hybrid composites.

The tensile properties of conducting polymers are very important. These properties might vary depending on the chosen processing technology, as different technologies (in our case, injection molding and 3D printing) produce different microstructures. Figure 6 shows the tensile test results for each composite. The tensile strength of the nanocomposites increased up to a CNT content of 0.75 wt% and then began to decrease. Tensile modulus and elongation at break remained almost constant regardless of reinforcement content. The reason for these phenomena is the aggregation tendency of CNTs—0.75 wt% of CNT was still able to disperse in the PLA matrix properly during compounding, but above this, dispersion was not sufficient. CNTs were then unable to produce their reinforcing effect, and the aggregates, which acted as stress concentrating centers, contributed to failure.

For composites reinforced with 30 wt% CF and CNT, tensile strength and modulus, and elongation at break decreased with increasing CNT content. This means that an increased CNT content makes the composite brittle. As a result, even smaller defect sites and aggregates were sufficient for the appearance of cracks, leading to failure. This may be the reason for the decrease in tensile strength and elongation at break.

#### 3.1.4 SEM investigation

**Figure 7:**
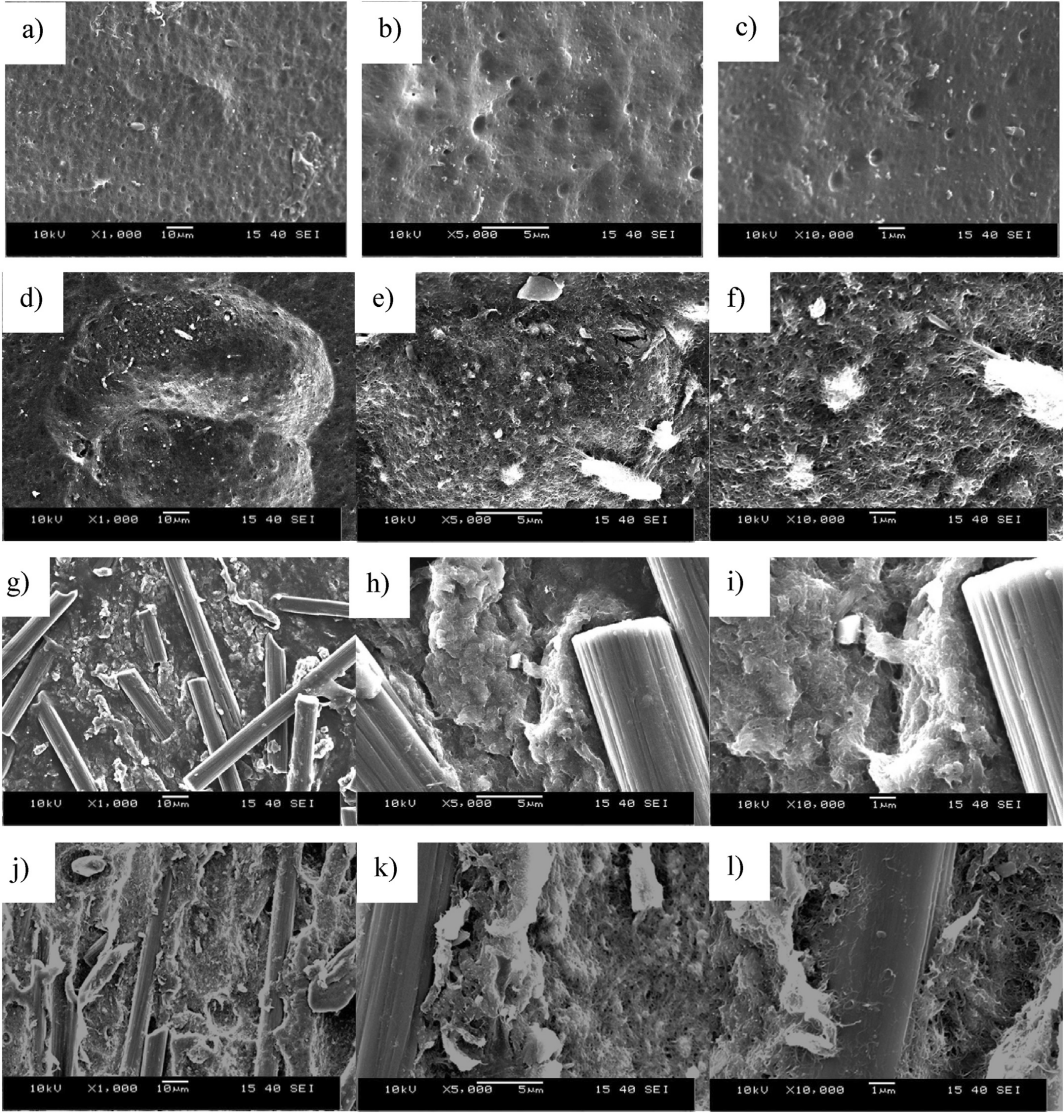
SEM images of a)-c) the injection-molded pure PLA, d)-e) the injection-molded PLA+0.5CNT nanocomposite, f)-i) the injection-molded PLA+30CF composite, j)-l) the injection-molded PLA+30CF+0.5CNT hybrid composite.

Figure 7 shows the SEM images of the injection molded samples. Large aggregates are visible on the SEM images of the PLA+0.5CNT nanocomposite and between the large aggregates, the few dispersed CNTs are not enough for good electrical connection. In the PLA+30CF composite, the carbon fibers are well dispersed and randomly oriented, causing them to cross each other, making electrically conductive pathways. In the hybrid composites, the well-dispersed nanotubes electrically contact the carbon fibers, increasing electric conductivity.

### 3.2 Increasing processability with OLA

When the OLA plasticizer was added to the hybrid composite reinforced with 30 wt% CF and 0.75 wt% CNT, MFI increased from 6.55 ± 0.7 to 19.2 ± 1.7 g/10 min, which is more than three times as much. It means that the plasticizer decreased the viscosity and acted as a slip additive inside the material, which facilitated the movement of the nano- and microparticles in the melt. The easier melt processability made it possible to process the composite via 3D printing. The OLA plasticizer was added in a second extrusion step to the hybrid composite, as the plasticizing effect of the OLA could have prevented the dispersion of the nanotubes.

#### 3.2.1 Electrical conductivity

**Figure 8:**
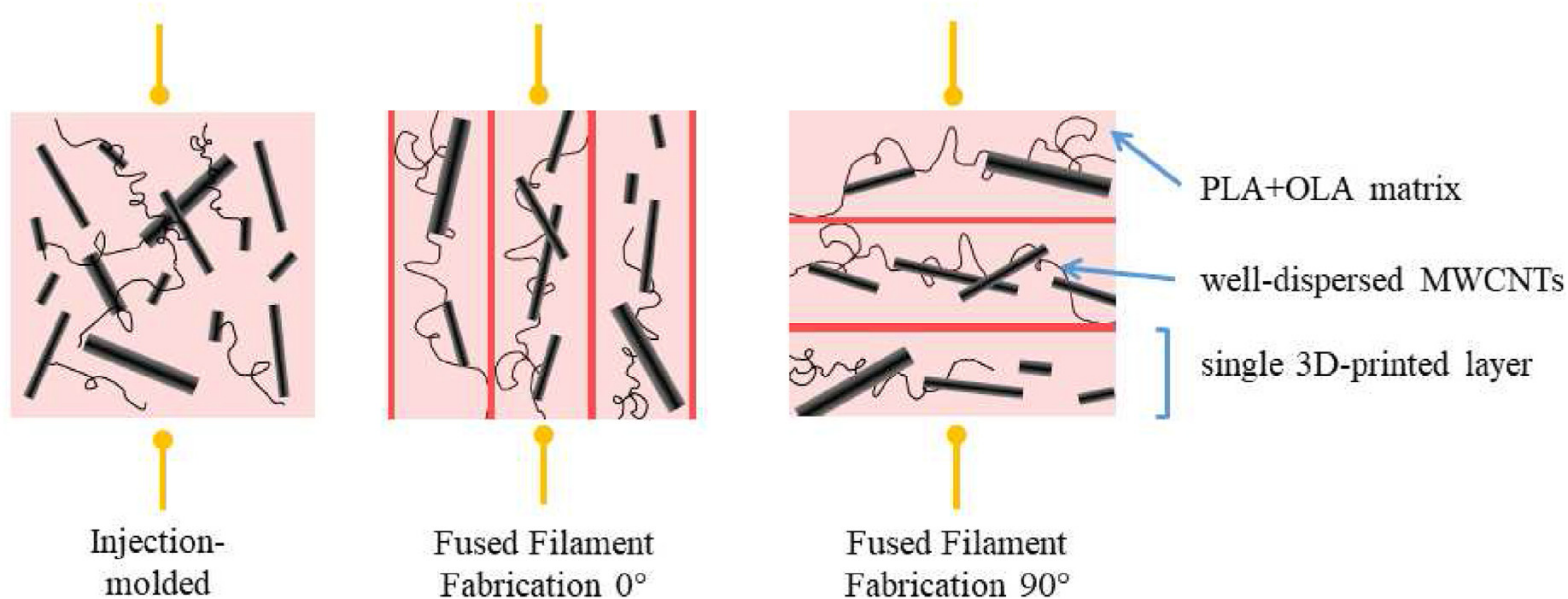
Schematics of the microstructure of the composites and the direction of the measurement of electrical conductivity.

We performed conductivity tests again to investigate the effects of plasticization. The plasticized hybrid composite was formed into a filament and was processable via 3D printing, therefore we were able to examine the effects of printing orientation. In material extrusion-based additive manufacturing, the direction of melt deposition aligns the fibers, thus the conductive paths as well, which is expected to cause changes in conductivity as a function of printing direction. Schematics of the hypothetical conductive paths can be seen in Figure 8. This also means that electrical conductivity can be tailored to demand within a single layer. The results in Table 2 show that in the case of the 3D printed samples, the electrical conductivity measured parallel to the printing direction (0°) is more than three times the conductivity measured perpendicular to it (90°), which meets our expectations and also aligns with the literature [30]. The difference in electrical conductivity between the injection molded and the 3D printed samples may be due to voids in the 3D printed structures.

**Table 2:** Electrical conductivity of the plasticized composite.

Name	Processing technology	Electrical conductivity (S/cm)
PLA+30CF+0.75CNT+10OLA	Injection molding	0.229
	Fused Filament Fabrication 0°	0.154
	Fused Filament Fabrication 90°	0.046

#### 3.2.2 Tensile properties

Table 3 shows the tensile mechanical properties of the plasticized hybrid composites. As the void content of the 3D-printed samples have a significant effect on mechanical behavior, we provide density-specific values [31]. When OLA2 was added to the PLA+30CF+0.75CNT composite, tensile strength, elongation at break, and tensile modulus became nearly the same as those of the 30CF only composite. This means that in the PLA+30CF+0.75CNT+10OLA composite, the plasticizer counteracted the embrittling effect of the carbon nanotubes, while not reducing electric conductivity compared to PLA+30CF+0.75CNT. This is of great importance as the embrittling effect of conductive additives have rarely been addressed in literature where most often, the elongation at break is reduced to about two-thirds [12].

**Table 3:** Tensile mechanical properties.

Manufacturing technology	Specific tensile strength (Nm/kg)	Tensile modulus (Nm/kg)	Elongation at break (%)
Injection molding	82.0 ± 2.8	8107.0 ± 741.5	1.21 ± 0.110
Additive manufacturing 0°	35.1 ± 4.6	10721.0 ± 1412.6	0.47 ± 0.510
Additive manufacturing 90°	23.5 ± 1.7	6530.4 ± 491.7	0.37 ± 0.001

#### 3.2.3 SEM investigation

**Figure 9:**
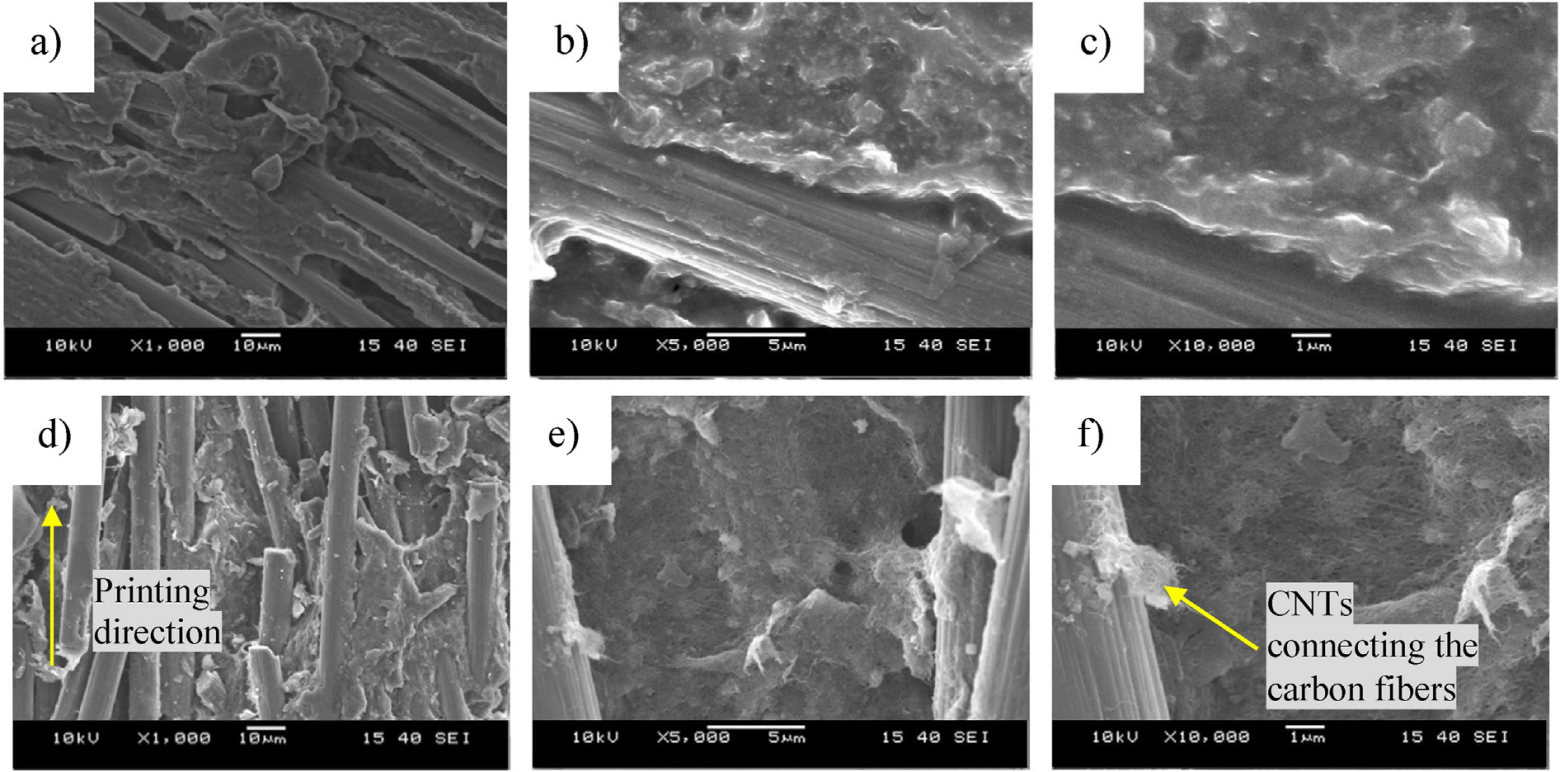
SEM images of a)-c) the injection molded and of d)-f) the 3D printed PLA+30CF+0.75CNT hybrid composite.

In the injection-molded samples (Figure 9 a.-c.), the fibers are randomly oriented and intersect at several points, creating an electrically conductive pathway. Similarly to the unplasticized sample, the carbon nanotubes are well dispersed, increasing the electric conductivity by making more electric connections between the carbon fibers. In the 3D printed samples (Figure 9 d.-f.), the carbon fibers are oriented in the printing direction so that they intersect at far fewer points and are less able to form a conductive network. The electrical connection caused by the dispersed CNTs between the carbon fibers provides good electrical conductivity even in highly oriented composites.

## 4 Conclusions

In this study, we developed electrically conductive nano- and hybrid composites with a poly(lactic acid) (PLA) matrix for different melt processing technologies. Electric conductivity only slightly increased when carbon nanotubes were added to the PLA matrix. When carbon fibers were added to the nanocomposites, the higher shear during melt mixing helped the uniform dispersion of the carbon nanotubes, which greatly increased the conductivity of the composite. On the other hand, the micro- and nanoscale hybrid reinforcement greatly increased viscosity, making melt processing difficult. The hybrid composite also became brittle, and the cracks in it propagated faster under a smaller load. This decreased tensile strength and elongation at break. Viscosity decreased when an oligomeric lactic acid plasticizer was added to the hybrid composites, resulting in easier processability either by injection molding or 3D printing. In addition, the composite became more ductile, the tensile strength and the elongation at break increased, while the electrical conductivity decreased only slightly.

## Declarations

### Author contribution statement

Roland Petrény: Conceived and designed the experiments; Performed the experiments; Analyzed and interpreted the data; Contributed reagents, materials, analysis tools or data; Wrote the paper.

Csenge Tóth: Performed the experiments; Analyzed and interpreted the data; Wrote the paper.

Aurél Horváth: Performed the experiments; Analyzed and interpreted the data.

László Mészáros: Conceived and designed the experiments; Wrote the paper.

### Funding statement

This work was supported by the National Research, Development and Innovation Office, Hungary (2018-1.3.1-VKE-2018-00001 and OTKA FK134336) and by the Italian-Hungarian bilateral agreement (grant number NKM-73/2019) of the Hungarian Academy of Sciences. The research reported in this paper is part of project no. BME-NVA-02, implemented with the support provided by the Ministry of Innovation and Technology of Hungary from the National Research, Development and Innovation Fund, financed under the TKP2021 funding scheme. László Mészáros is thankful for János Bolyai Research Scholarship of the Hungarian Academy of Sciences, and for the ÚNKP-21-5 New National Excellence Program of the Ministry for Innovation and Technology.

### Data availability statement

Data included in article/supplementary material/referenced in article.

### Declaration of interests statement

The authors declare no conflict of interest.

### Additional information

No additional information is available for this paper.
